# Development of an optically emulated computed tomography scanner for college education

**DOI:** 10.1186/s42492-025-00211-z

**Published:** 2026-01-16

**Authors:** Md. Motaleb Hossen Manik, William Muldowney, Md. Zabirul Islam, Ge Wang

**Affiliations:** 1 Department of Computer Science, Rensselaer Polytechnic Institute, Troy, NY 12180 United States; 2 Department of Biomedical Engineering, Rensselaer Polytechnic Institute, Troy, NY 12180 United States

**Keywords:** Optical computed tomography, Visible light imaging, Low-cost imaging systems, Educational imaging, Visual computing

## Abstract

Computed tomography (CT) is a powerful imaging modality widely used in medicine, research, and industry for noninvasive visualization of internal structures. However, conventional CT systems rely on X-rays, which involve radiation exposure, high equipment costs, and complex regulatory requirements, making them unsuitable for educational or low-resource settings. To address these limitations, we developed a compact, low-cost, optically emulated CT scanner that uses visible light to image semi-transparent specimens. The system consists of a rotating stage enclosed within a light-isolated box, backlight illumination, and a fixed digital single-lens reflex camera. A Teensy 2.0 microcontroller regulates the rotation of the stage, while MATLAB is used to process the captured images using the inverse Radon transform and visualize the reconstructed volume using the Volumetric 3D MATLAB toolbox. Experimental results using a lemon slice demonstrate that the scanner can resolve internal features such as the peel, pulp, and seeds in both 2D and 3D renderings. This system offers a safe and affordable platform for demonstrating CT principles, with potential applications in education, industrial inspection, and visual computing.

## Introduction

Computed tomography (CT) is a widely used imaging modality that enables non-destructive visualization of internal structures in both biological and non-biological specimens. Its applications span across fields such as diagnostic medicine, industrial inspection, and digital preservation in the arts. Despite its utility, traditional CT systems depend on ionizing X-rays, which require costly shielding infrastructure, trained personnel, and regulatory approval. These constraints make conventional CT systems largely inaccessible in educational settings, low-resource environments, and public demonstrations where safety, affordability, and portability are critical.

To bridge this gap, we present a compact optically emulated computed tomography (OECT) scanner that employs visible light as the imaging medium. Unlike X-ray CT, our system operates safely without radiation hazards, allowing for interactive use in classrooms, workshops, or informal learning spaces. The device is designed to visualize semi-transparent specimens–such as fruit slices or plant samples–by capturing transmitted light from multiple angles and reconstructing internal structures using tomographic techniques.

The scanner employs a fixed imaging setup consisting of a digital single-lens reflex (DSLR) camera with a prime lens, a battery-operated light emitting diode (LED) backlight, and a custom-fabricated light box that eliminates ambient light interference. The specimen is mounted on a PLA 3D-printed rotating stage powered by a stepper motor, and its motion is regulated using a Teensy 2.0 Arduino-compatible microcontroller. Sequential images are acquired at regular angular intervals as the specimen rotates. These images are then processed in MATLAB to generate sinograms, perform inverse Radon transformations, and construct volumetric 3D visualizations using the Vol3D rendering toolbox.

Our system builds upon and extends prior work in optical tomography and educational imaging platforms. Optical CT has been explored in diverse applications, ranging from education to radiotherapy verification, highlighting its potential as a safe, non-ionizing imaging modality. While these various applications demonstrate the versatility of optical CT, our approach specifically targets educational accessibility by integrating consumer-grade components with a complete, reproducible imaging pipeline.

For example, Lim et al. [1] designed an educational optical CT scanner prototype using visible light, a DSLR camera, and an Arduino-based motor controller. Their low-cost design, featuring a plywood lightbox and 3D-printed components, successfully reconstructed 3D volumes in MATLAB, proving its effectiveness for teaching CT principles without radiation risks.

Zakariaee et al. [2] developed an optical CT system for polymer gel dosimetry in radiotherapy verification. Using a He-Ne laser source, photocell detector, and custom-built motion stages, the system achieved sub-millimeter spatial resolution and high uniformity. This work demonstrates how optical CT can be adapted for high-precision, three-dimensional dose verification.

Kang et al. [3] presented a differentiable, dynamic visible-light tomography framework that combined multiplexed acquisition with deep learning–based reconstruction. The system enabled real-time 3D capture of dynamic scenes at 60 frames per second, illustrating how optical CT can be extended to time-resolved imaging applications.

Optical projection tomography (OPT) is another visible-light-based tomographic technique commonly used for high-resolution 3D imaging of small, transparent biological samples [4]. Traditional OPT systems require immersion in index-matching liquid to minimize refraction artifacts at the specimen boundaries. Liang [5] proposed a method to eliminate the need for such immersion by incorporating ray-tracing corrections based on a known surface model. In a different direction, Stavroulakis et al. [6] demonstrated that OPT can be successfully applied to large, thin-walled transparent objects such as glassware and plastic bottles even without refractive index matching, enabling broader applications in industrial and cultural heritage imaging.

Rubinoff et al. [7] introduced adaptive spectroscopic visible-light OCT for clinical retinal oximetry, explicitly removing spectral contaminants (e.g., spectrally dependent roll-off, spectrally dependent background bias, and longitudinal chromatic aberration) via depth-resolved STFT normalization and vessel-specific parameter selection. Ex vivo and in vivo validation (18 volunteers) showed accurate and repeatable oxygen saturation estimates (RMSE $\approx$ 2.1% *vs* pulse oximetry in major arteries; repeatability $\approx$ 2.2%–2.5% across vessels). Although focused on ophthalmic oximetry, this work exemplifies how adaptive, model-based correction and depth selection in non-ionizing optical CT can improve quantitative reliability, offering transferable lessons for optical CT acquisition and processing.

Low-cost imaging systems demonstrate how modularity, affordability, and open-source design can broaden access to advanced imaging technologies for both education and prototyping.

Jonveaux [8] introduced an Arduino compatible development kit for single-element ultrasound imaging. The platform integrated open-source hardware, firmware, and modular electronics, enabling students and researchers to explore imaging concepts at low cost. Although ultrasound-based, this work exemplifies how inexpensive, replicable hardware can support learning goals similar to those of optical CT.

Shu et al. [9] developed a portable visible-light optical coherence tomography (vis-OCT) system for clinical retinal imaging. By using shorter illumination wavelengths, it achieved sub-micrometer resolution and improved scattering contrast. It highlights how compact, non-ionizing optical systems can be adapted for specialized imaging tasks, inspiring educational uses of optical CT.

Wang et al. [10] introduced a dual-channel vis-OCT system that achieves 1.3 $\upmu$m axial resolution and wide-field retinal imaging by combining a linear-in-*k* spectrometer, pathlength modulation, and noise cancellation. The system supports Doppler and angiographic modes, advancing clinical applications of vis-OCT in ophthalmology.

Reconstruction methods are critical for transforming projection data into accurate tomographic images, particularly when sampling is sparse or computational efficiency is required.

Shih et al. [11] applied the algebraic reconstruction technique (ART) to optical CT of polymer gel dosimeters. Compared to filtered backprojection (FBP), ART reduced streak artifacts and improved spatial accuracy under limited-angle conditions, showing the benefits of iterative methods for optical CT.

Fan and Zhu [12] proposed an ultrasonic transmitted-wave CT system for nondestructive inspection of concrete structures. Using interpolation and a modified maximum likelihood expectation maximization algorithm, it improved defect localization and reduced artifacts. Though developed for ultrasound, the reconstruction principles are relevant to optical CT under sparse sampling.

Zeng [13] introduced a displacement function based interpolation technique for sparse-view tomography. By synthesizing intermediate sinogram projections, the method preserved structural details and reduced blurring compared to conventional interpolation. This approach provides insight for optical CT when projection numbers are limited.

Zeng et al. [14] developed a three-dimensional backprojection filtering algorithm for time-of-flight positron emission tomography. By incorporating a time of flight-modified ramp filter, the method improved spatial resolution and noise suppression while maintaining analytic speed. These principles can inspire more efficient reconstruction pipelines in optical CT.

Advanced rendering methods enhance the interpretability of CT datasets, offering improved realism, spatial clarity, and educational value. Glemser et al. [15] applied cinematic volume rendering to clinical CT data, simulating realistic light interactions with volumetric datasets. Their approach improved anatomical depth perception and interpretability compared to conventional rendering. Such techniques can be adapted to optical CT to enrich the visualization of semi-transparent structures.

Wallner-Essl et al. [16] explored 3D CT cinematic rendering (CR) for forensic skeletal trauma analysis. CR produced photorealistic images with enhanced fracture visibility and clearer spatial relationships, improving both expert interpretation and courtroom communication. While computationally intensive, this method highlights how advanced rendering could similarly transform optical CT visualization.

Böttcher et al. [17] explored 3D CR of cardiovascular CT data in augmented reality using HoloLens devices. Radiologists and cardiologists rated the visualizations, intuitive and helpful for anatomical understanding. While direct clinical use was limited, the approach showed strong potential for education, patient communication, and interdisciplinary collaboration.

Unlike prior open-source or do-it-yourself scanner projects that primarily emphasize on mechanical prototyping or single-view scanning, our implementation integrates mechanical design, electronics, optics, and image processing into a cohesive, reproducible system. It offers a hands-on introduction to the principles of tomography while demonstrating practical skills in optics, embedded systems, and signal processing.

In this paper, we describe the system architecture, operational workflow, image reconstruction pipeline, and experimental validation using a lemon slice as the test object. We also analyze the system’s current limitations and explore its potential applications in education, research, and visual computing contexts.

## Methods

This section outlines the methodology for designing, building, and evaluating an OECT scanner, including the system architecture, scanning protocol, and image processing for tomographic reconstruction and visualization.

### System design and components

The OECT scanner was designed as a modular, desktop-scale system, comprising four core subsystems: a light box and illumination unit, a rotating stage mechanism, an optical imaging setup, and control circuitry. Each subsystem was carefully selected and assembled, considering accessibility, cost, and reproducibility, which enabled the construction of the scanner using consumer-grade components, 3D-printed parts, and open-source tools.

**Fig. 1:**
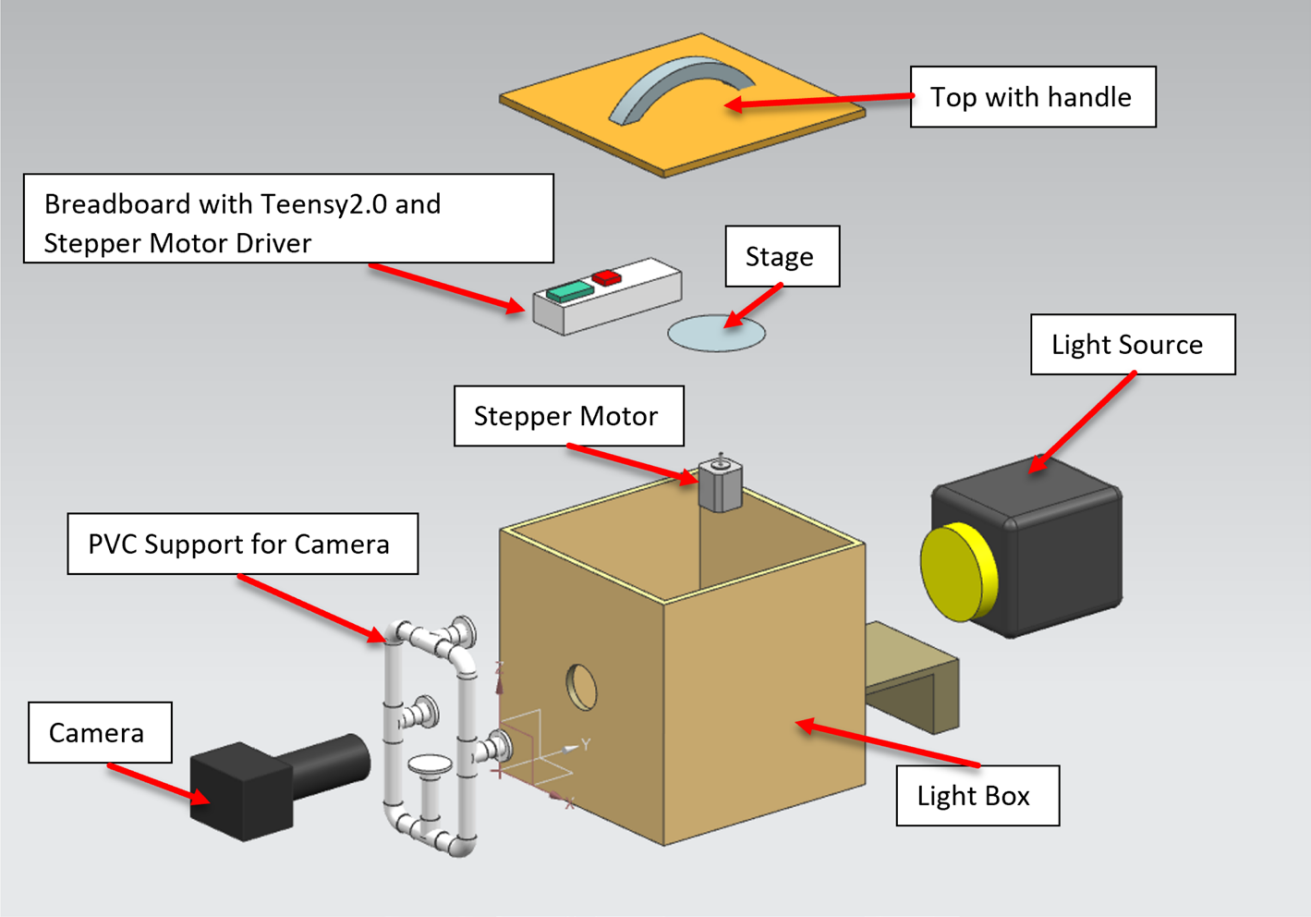
Design of the system

The system was designed to minimize the external interference and maximize the contrast and clarity of the acquired transmission images. The modular design also allows for easy troubleshooting, upgrading, and adaptation to other imaging scenarios. The overall hardware setup is shown in Fig. 1.

#### Light box and illumination

A critical requirement for an effective OECT is minimizing the impact of ambient light and achieving uniform illumination across the specimen. To this end, a custom light box was constructed from plywood chosen for its affordability, rigidity, and ease of machining. The box was designed as a fully rectangular enclosure with a fixed base and a detachable top lid to allow specimen loading and hardware access.

To suppress internal reflections and prevent light contamination, the inner surfaces of the light boxes were painted matte black. Strategically cut apertures were made on the sides to accommodate the camera lens, a universal serial bus (USB) cable for powering and programming the motor controller, and a physical toggle switch for motor operation.

A midwall was added inside the box to serve as a mounting plane for the illumination source. A Milwaukee LED light bar (dimensions: 12 cm $\times$ 2.5 cm $\times$ 2.5 cm) was affixed to the midwall using its built-in magnetic backing. This configuration allows quick installation and alignment. A square-frame diffuser was placed in front of the LED to scatter light evenly and eliminate harsh shadows, producing uniform backlighting across the specimen area.

The illumination source operates on an internal battery, enabling the system to function without tethered power cables, an important design consideration for safety, mobility, and ease of use in classroom environments. The combination of controlled backlighting and light isolation provided by the enclosure ensured that the camera captured high-contrast images with minimal external noise. This configuration ensures that the specimen is evenly illuminated, centrally positioned within the optical path, and shielded from ambient light–factors critical for consistent image quality.

**Fig. 2:**
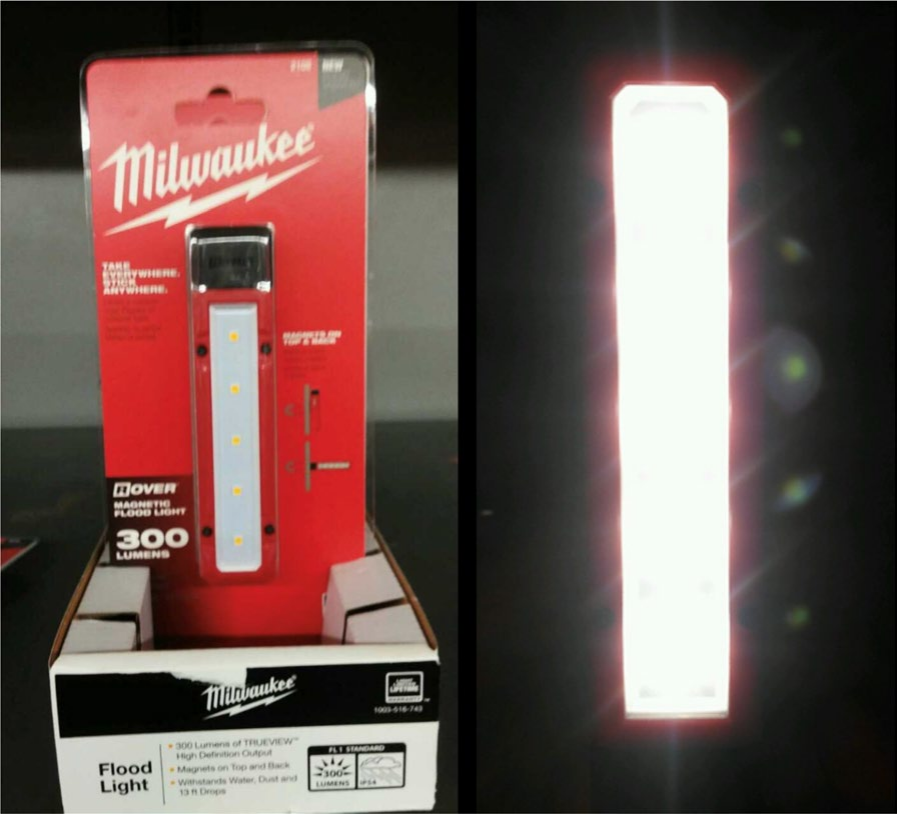
Light source

The LED backlight and diffuser configurations are shown in Fig. 2.

#### Rotating stage mechanism

Instead of rotating the imaging apparatus around the specimen, as is common in clinical CT systems, the desktop optical scanner employs a rotating stage to turn the specimen while the imaging components remain fixed. This approach reduces the mechanical complexity and implementation cost.

**Fig. 3:**
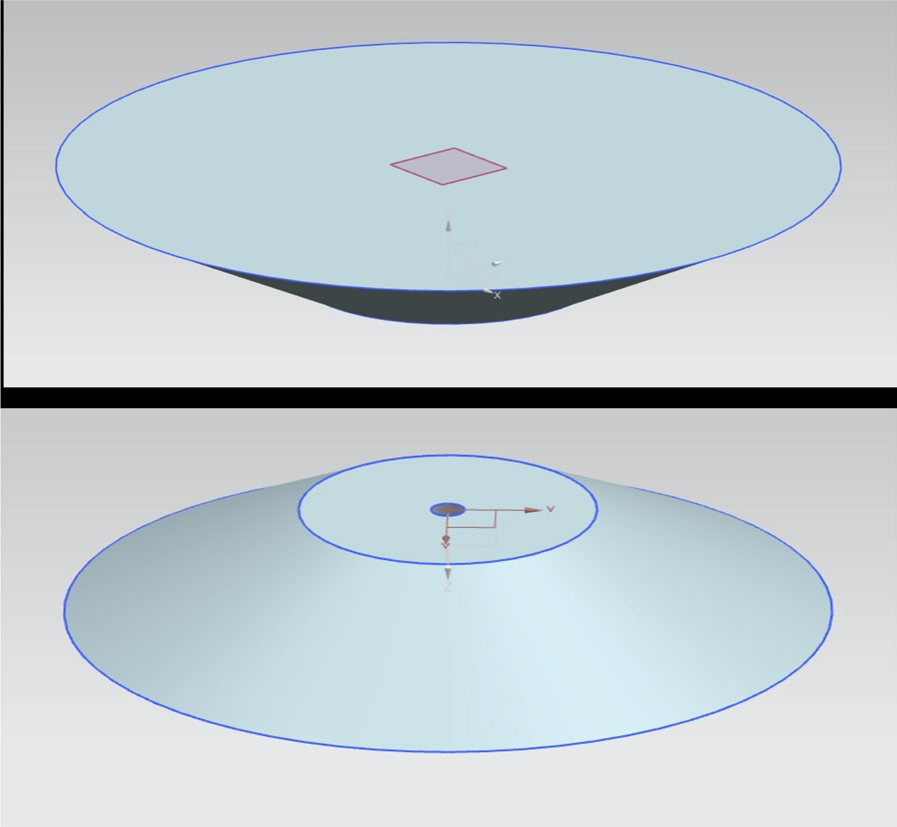
Rotating stage - 3D printed part

**Fig. 4:**
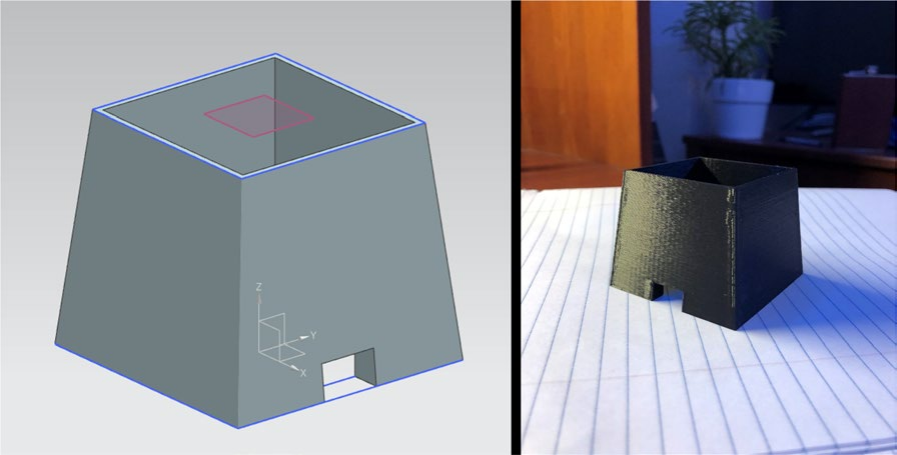
3D printed motor holder

The rotating platform and its supporting motor holder were fabricated using fused deposition modeling 3D printing technology. The material used was polylactic acid (PLA), a commonly available biodegradable thermoplastic known for its dimensional stability, workability, and low cost. After printing, the parts were lightly sanded and drilled to correct small dimensional inaccuracies and ensure a precise fit with the stepper motor shaft. Figure 3 shows the 3D printed rotating platform, and the motor holder and its housing are shown in Fig. 4.

The specimen was mounted directly on a rotating stage, which was driven by a stepper motor positioned inside a light box. To reduce movement artifacts during scanning, a strip of paper towel was placed between the base of the specimen and the stage platform to increase friction and prevent slippage during rotation. This simple but effective modification improved stability without requiring adhesives or complex mounting mechanisms.

The motor and stage assemblies were carefully positioned in front of the backlight to ensure that the specimen remained centered and fully illuminated throughout the scan. This alignment is critical for an accurate sinogram formation and successful tomographic reconstruction.

#### Optical imaging unit

Accurate OECT reconstruction relies on the consistent acquisition of high-quality images at regular angular intervals. The scanner employed was a Nikon D5600 DSLR camera paired with a 50 mm f/1.8 prime lens. This combination was selected to balance the image resolution, light sensitivity, affordability, and ease of integration.

The Nikon D5600 DSLR camera offers a high-resolution Advanced Photo System type-C sensor and full manual control over the exposure and focus parameters–features essential for maintaining consistency across all captured projection images. The 50 mm prime lens provides a wide aperture (f/1.8), which increases the camera’s ability to capture sufficient light under low-light conditions inside the sealed light box. Moreover, prime lenses produce minimal distortion and high image sharpness, making them suitable for tomographic applications where geometric accuracy is critical.

The camera was mounted onto a custom-stabilizing apparatus to ensure repeatable positioning and minimize motion artifacts. This apparatus was constructed from lightweight PVC pipes, fitted with three 3D printed pipe connectors designed by Siemens NX and fabricated using PLA. Pipe fitters allow fine control over the alignment and elevation of the camera relative to the lightbox.

The entire imaging rig was securely attached to the external side of the light box with the lens aligned precisely through a circular aperture. This aperture was carefully positioned to align the camera’s optical axis with the rotational center of the specimen. This alignment ensures that each captured image corresponds to a consistent radial viewpoint, minimizing distortion and enabling accurate sinogram construction.

**Fig. 5:**
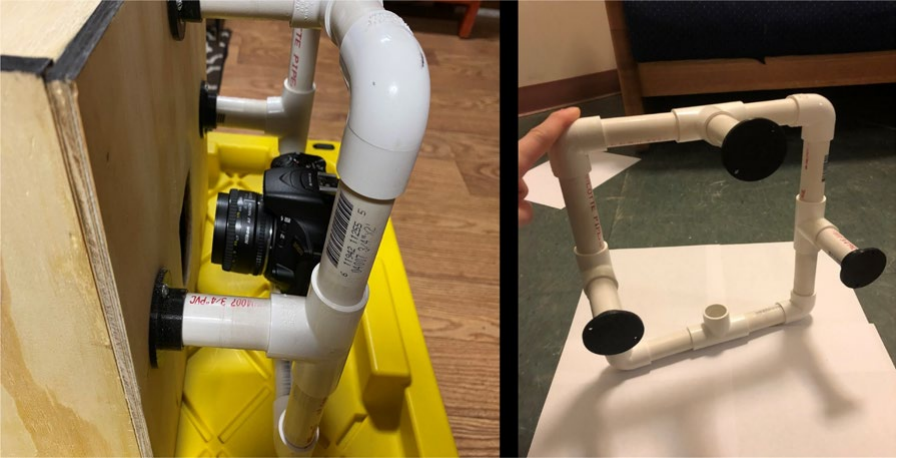
Camera apparatus mounted to the light box with 3D printed stabilizers

The use of a consumer-grade DSLR camera along with the modular mounting design contributes to the system’s overall accessibility and replicability, allowing educators and students to reproduce the setup without relying on proprietary or specialized hardware. The camera setup is illustrated in Fig. 5.

#### Control circuitry and electronics

The rotational motion of the specimen stage was controlled using a stepper motor regulated by a simple yet effective electronic circuit assembled on a solderless breadboard. This modular configuration enables the easy testing, reconfiguration, and educational demonstration of embedded control systems.

The core of the control circuit is a Teensy 2.0 microcontroller board chosen for its compact form factor, Arduino IDE compatibility, and affordability. It serves as a controller for regulating motor step timing and direction, providing precise incremental movement synchronized with the image acquisition process. Its compatibility with standard Arduino libraries simplifies programming and renders the platform accessible to students and hobbyists.

The stepper motor is driven by a dedicated stepper motor driver module embedded within the breadboard circuit. The driver is equipped with a heat sink to dissipate the thermal load during continuous operation, ensuring consistent performance and preventing thermal shutdown. The use of a stepper motor, rather than a servo or DC motor, allows for precise angular control without the need for feedback systems, an important feature of tomography, where projection angles should be evenly spaced.

All the circuit components were connected using jumper wires, enabling rapid prototyping and a non-permanent configuration. This approach supports experimentation and modification without soldering, making it ideal for classroom and laboratory environments.

A toggle switch mounted on the exterior of the light box provided manual on/off control of the motor circuit. The Teensy board was powered by a USB connection to an external computer, which serves as a platform for programming the microcontroller and storing captured image data.

**Fig. 6:**
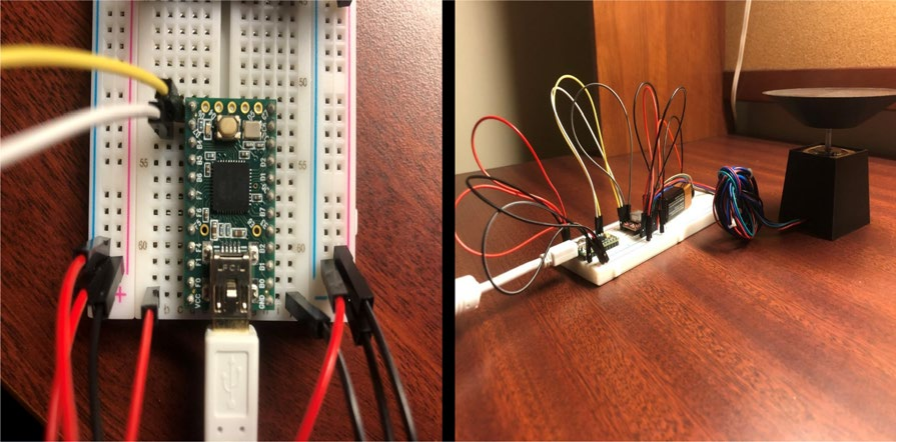
Breadboard circuit with Teensy 2.0, stepper motor driver, and power switch

This combination of accessible microcontroller technology and reconfigurable circuitry provides a flexible and low-cost control system for operating the scanner’s mechanical components reliably and precisely. The control setup is illustrated in Fig. 6.

#### Integration and reproducibility

The OECT scanner was intentionally designed as a modular, low-cost platform, in which each subsystem–illumination, rotation, imaging, and control–was built using accessible materials and tools. This modularity ensures straightforward assembly, ease of debugging, and flexibility for customization by students or educators.

All components were either off-the-shelf, 3D-printable, or programmable, with widely available microcontrollers. This enabled the system to be easily replicated without specialized facilities. Table 1 summarizes the key components and their estimated costs, and Table 3 in Appendix B lists the detailed materials used in the OECT scanner.

**Table 1:** Integrated summary of components with estimated cost and function

Component	Function	Cost (USD)
Plywood sheets	Encloses the system and blocks ambient light	$10
Milwaukee LED light bar	Provides uniform backlight illumination	$25
Diffusor panel	Spreads light evenly to reduce shadows	$5
PLA filament	Used for 3D printed stage, rig, and mounts	$15
NEMA 17 stepper motor	Drives precise rotation of specimen	$12
A4988 motor driver	Regulates stepper motor current	$3
Teensy 2.0 microcontroller	Generates motor control signals	$20
Breadboard and jumper wires	Circuit prototyping without soldering	$8
Nikon D5600 DSLR camera	Captures high-resolution projection images	$500
50 mm f/1.8 prime lens	Enhances sharpness and low-light capture	$100
Miscellaneous (tape, cork, toothpicks, etc.)	For mounting and specimen support	$7
**Total estimated cost**		**$705**

This configuration offers a balance between the imaging performance and affordability, particularly in classrooms. Users may substitute lower-cost alternatives, such as webcam-based imaging systems, although this may reduce resolution and optical fidelity. This modular structure encourages experimentation and learning in the fields of optics, electronics, and image reconstruction.

### Scanning protocol

Successful tomographic reconstruction requires high-quality projection images to be captured at consistent angular intervals as the specimen rotates. To achieve this, the scanner should be operated using a carefully controlled step-by-step workflow. This subsection describes the complete process of preparing the specimens, configuring the system, initiating image capture, and formatting the resulting data.

#### Specimen mounting and setup

To ensure optimal image quality and consistent reconstruction, the specimen should be properly prepared, and all subsystems–lighting, camera, and motor–must be carefully configured. This subsection describes the integrated procedure used to mount the specimens and prepare the scanning system.

#### Specimen selection and mounting

The OECT scanner was designed to image semi-transparent objects such as citrus slices, soft plant tissues, or gel phantoms. These specimens allowed visible-light transmission while revealing their internal structures.

1. **Select a suitable specimen:** Use a thin and relatively flat object, such as a lemon slice approximately 5–8 mm thick. Thinner samples reduce scattering and enhance contrast.
2. **Mount the specimen:** Insert a toothpick or slender nail vertically into a cork base to serve as a support. Gently pierce the specimen onto the post so it stands upright.
3. **Stabilize on the stage:** Secure the cork base to the center of the rotating stage using electrical tape or a similar low-profile adhesive. Ensure the specimen is vertical and centered to avoid motion artifacts during scanning.

#### Motor and circuit configuration

The rotation stage was powered by a stepper motor controlled by a Teensy 2.0 microcontroller. Before scanning:

1. Confirm jumper wires and breadboard connections follow the finalized schematic.
2. Check that the motor driver and its heat sink are securely mounted.
3. Connect the Teensy 2.0 board via USB to a computer; the onboard LED should glow orange when powered.

#### Lighting configuration

Uniform backlighting and ambient-light shielding are critical for transmission imaging.

1. Activate the Milwaukee LED light using its power button.
2. Mount the square-frame diffusor onto the light using the mid-wall fixtures.
3. Position the stage and motor assembly in front of the diffusor without direct contact.
4. Close the light box lid fully to block external light, as discussed in Light box and illumination subsection.

#### Camera setup

Consistent image-capture settings are essential for accurate sinogram generation.

1. Set the Nikon D5600 DSLR camera to Program (P) mode.
2. Adjust manual focus to ensure a sharp central image.
3. Disable live view to conserve battery.
4. Navigate to the Shooting Menu and activate the Interval Timer Shooting.
5. Set the interval to 1 second with 200 total frames.
6. Start image capture only after activating the motor to maintain synchronization.

This streamlined workflow balances the manual and automated steps to prepare the specimen and system for efficient, high-fidelity OECT scanning.

#### Data acquisition

Once the system is fully configured and the specimen is correctly mounted, as described in Light box and illumination subsection, the image acquisition can begin. The timing between the motor rotation and the camera shutter should be closely coordinated to ensure that each captured frame corresponds to a distinct projection angle.

1. Flip the red toggle switch located on the side panel of the light box to activate the stepper motor and initiate stage rotation.
2. Immediately after the specimen begins rotating, press Start on the Nikon D5600 DSLR camera’s interval timer to begin automated image capture.
3. The scanning session proceeds automatically, with the camera capturing one image per second for a total of 200 frames–completing a full 360-degree sweep.
4. Once all images have been captured and stored to the SD card, flip the toggle switch to the OFF position to stop the motor and prevent overheating.

This coordinated acquisition method ensures that each projection corresponds to a unique rotational angle, thereby forming the complete dataset required for sinogram generation and tomographic reconstruction.

#### Image handling and preprocessing

After scanning, raw image data should be transferred, cleaned, and formatted for computational processing. This preparation step is essential to ensure efficient memory usage and isolate the region of interest from the background elements.

1. Remove the SD card from the camera and insert it into a computer using a card reader or USB adapter.
2. Copy the 200 captured images into a dedicated folder and rename them sequentially (e.g., I (1), I (2),..., I (200)) using a bulk renaming tool or file manager.
3. Use a batch image resizer to uniformly reduce the resolution of the images to match the available system memory during MATLAB processing.
4. Apply bulk cropping to each image to isolate the specimen in the center of the frame and eliminate extraneous background features that could interfere with reconstruction accuracy.

At the end of this step, the dataset consisted of 200 size-optimized, uniformly cropped grayscale images, each representing a projection from a unique angle. These images served as inputs to the MATLAB-based reconstruction pipeline described in the next section.

### Image processing and 3D reconstruction

After completing the scanning protocol and preparing the acquired image dataset, the next phase involved converting the 2D projections into interpretable tomographic slices and volumetric visualizations. All image processing and reconstruction were conducted in MATLAB, which was chosen for its powerful matrix manipulation capabilities, built-in support for tomographic operations, and compatibility with open-source 3D visualization toolkits.

The reconstruction workflow consists of three primary steps: preprocessing the image stack, constructing a sinogram, and applying the inverse Radon transform, followed by 3D volume rendering. These steps are described in detail below:

#### Image preprocessing

Raw projection images should first be cleaned and standardized to ensure numerical stability and alignment throughout the reconstruction process. The key preprocessing steps are as follows:

1. **Transfer and rename:** All image files are copied from the camera’s SD card to a local directory and renamed sequentially using bulk file renaming tools. This ensures that the images are ordered by acquisition angle, typically as I (1), I (2),..., I (200).
2. **Resize:** Images are uniformly resized using a batch resizer tool to ensure that the total dataset fits within the available system random access memory, especially when generating 3D matrices or running real-time rendering in MATLAB.
3. **Cropping:** Bulk cropping is applied to all images to isolate the rotating specimen at the center of the frame, removing excess background regions that can introduce noise or reduce contrast in the sinogram.

These operations standardize the image stack and prepare the data for sinogram construction, a critical intermediate representation of tomographic imaging.

#### Sinogram generation

In optical CT, the sinogram is a key intermediate representation that encodes how internal structures attenuate light across multiple projection angles. Each column in the sinogram corresponds to the pixel intensities along the vertical slice of a single-projection image, and the rows track the evolution of the profile as the specimen rotates.

To generate the sinogram, all projection images are first converted to grayscale, resized for consistency, and stacked column-wise using a defined vertical slice (e.g., central column). The following MATLAB snippet demonstrates this process:
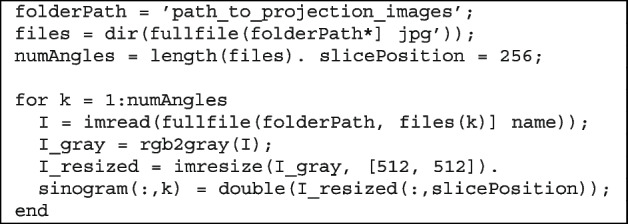


This process constructs a two-dimensional matrix where each column represents the light attenuation profile at different rotation angles. Consistent preprocessing, such as resizing and grayscale conversion, ensures that the sinogram reflects the true spatial variation rather than artifacts caused by inconsistent image sizes or lighting.

As the sinogram is the input to the inverse Radon transform, its quality directly affects the accuracy of the final tomographic reconstruction. Misalignment, shadowing, or uneven illumination appear as distortions or smearing in the reconstructed cross-section. Therefore, careful alignment and lighting configurations are critical in the early stages of the imaging pipeline.

#### Reconstruction using inverse Radon transform

Once the sinogram is generated, the next step is to reconstruct a 2D cross-sectional image of the specimen. This is accomplished by using the inverse Radon transform, which mathematically reverses the projection process by integrating the projection angles to recover the internal spatial structure.
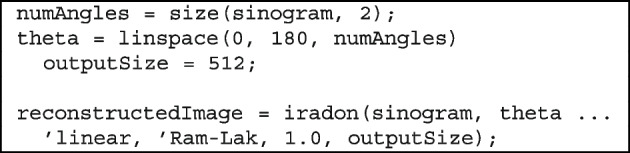


In MATLAB, this is typically implemented using the built-in iradon () function, which applies a FBP. The above code illustrates the reconstruction process.

Here, theta defines a set of projection angles evenly spaced between 0$^{\circ }$ and 180$^{\circ }$. The iradon () function applies the ’Ram-Lak’ filter to suppress low-frequency artifacts and improve edge sharpness.

The output is a 2D grayscale image revealing the internal composition of the scanned object. In this image, darker intensities correspond to regions of higher optical density (e.g., seeds or rinds), which block more light and therefore appear darker in the projection data.

This reconstruction technique provides a fast and effective method for visualizing the cross-sections of transparent or semi-transparent specimens, making it ideal for real-time feedback in educational or demonstration environments.

#### Volumetric rendering with Vol3D

To visualize the internal structure of the scanned object in three dimensions, the reconstructed 2D slices were stacked into a volumetric dataset and rendered using the open-source Vol3D toolbox in MATLAB.

The following code snippet shows a typical volumetric rendering workflow.
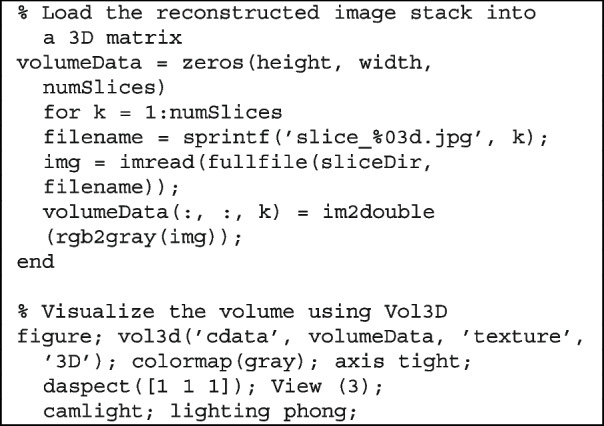


In this example, volumeData is constructed by loading grayscale slice images from a specified directory. Each image represents a reconstructed 2D cross-section of the specimen. The stack is passed to Vol3D (), enabling interactive exploration of the internal volume through GPU-accelerated rendering.

Adjusting the transparency and lighting settings enhances the contrast between different structural regions, such as the pulp, peel, and seeds. Users can interactively rotate, zoom in, and slice the volume for inspection from various angles.

This 3D rendering pipeline transforms raw slice data into intuitive and informative visualizations, which are ideal for both teaching and qualitative analysis of semi-transparent specimens.

**Fig. 7:**
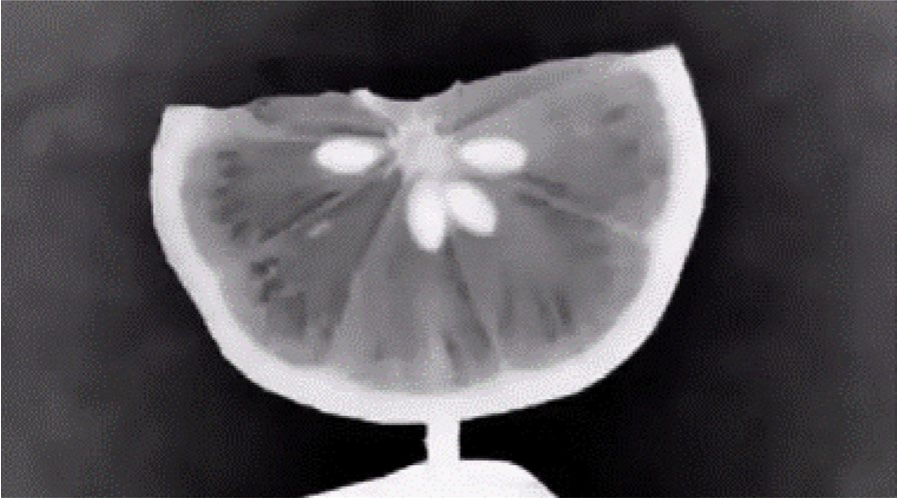
3D reconstruction of a lemon slice using Vol3D. Seeds and internal peel structure are clearly visible

This is visualized in the final 3D rendering shown in Fig. 7.

## Results

To assess the functionality and reconstruction fidelity of the desktop OECT scanner, a test scan was performed using a fresh lemon slice as a specimen. This object was selected for its partial transparency under visible light and its distinct internal features, such as seeds, pulp, and rind, which make it well-suited for evaluating transmission contrast and structural reconstruction.

### Projection image acquisition

Following the setup described in Results and Discussion sections, a complete scan was conducted by capturing 200 projection images as the specimen underwent a full 360-degree rotation. The Nikon D5600 DSLR camera, equipped with a 50 mm prime lens, successfully recorded high-resolution images at 1-s intervals using the camera’s interval timer.

The combination of backlighting and diffusion produced uniform illumination across the field of view, effectively highlighting the internal density variations within the lemon slice. The prime lens enables sharp contrast at low exposure levels, minimizing motion blur while preserving edge detail.

**Fig. 8:**
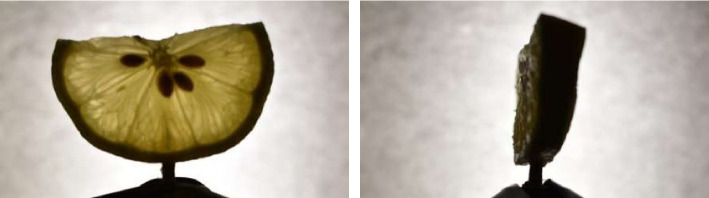
Example projection images of the lemon slice captured during the scan. Left: frontal view. Right: angled view showing light transmission through pulp and peel

These projections form the foundational dataset for generating the sinogram and subsequent tomographic reconstruction, as illustrated in the projection images shown in Fig. 8.

### Sinogram output

Using the preprocessed projection images, a sinogram was constructed by extracting a fixed vertical column of intensity values from the center of each image. This technique effectively maps the light attenuation profile of a specimen across its full rotational cycle.

**Fig. 9:**
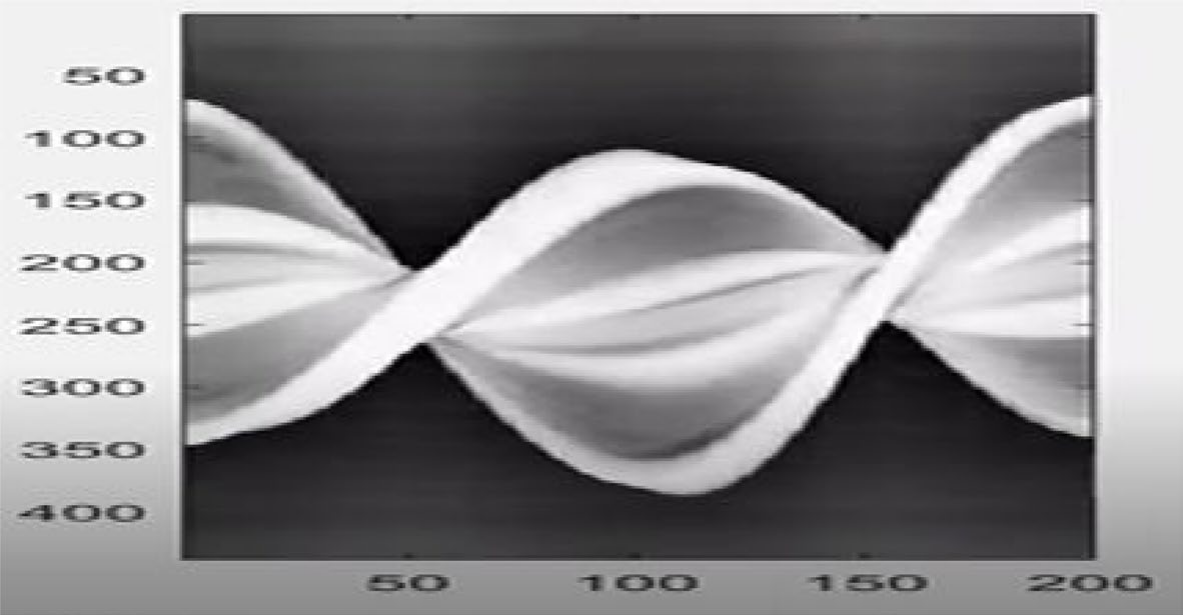
Sinogram generated from projection images. Repeating features reflect the rotational symmetry and internal structure of the lemon slice

The resulting sinogram revealed periodic features corresponding to the radial symmetry of the lemon slice. High-attenuation regions such as dense peels and embedded seeds appear as repeating vertical bands owing to their consistent opacity across rotation angles. Conversely, the pulp regions displayed smoother intensity variations, reflecting lower light attenuation. The corresponding sinogram is shown in Fig. 9.

The structure and quality of the sinogram directly influence the resolution and accuracy of the reconstructed cross-sectional images, as discussed in the following sections.

### Reconstruction results

To convert the projection data into a spatial cross-section, the inverse Radon transform was applied to the sinogram using MATLAB’s iradon () function. The resulting 2D grayscale image represents a slice reconstructed through the center of the lemon slice, with pixel intensities corresponding to local variations in light attenuation.

The reconstruction accurately preserved the specimen’s distinct anatomical features.

- **Outer peel:** Appears as a dark ring due to its high density, which causes strong attenuation of transmitted light.
- **Pulp region:** Brighter and more translucent, reflecting lower density and higher light transmission.
- **Seeds:** Visible as small dark spots near the center, corresponding to compact, highly attenuating internal features.

**Fig. 10:**
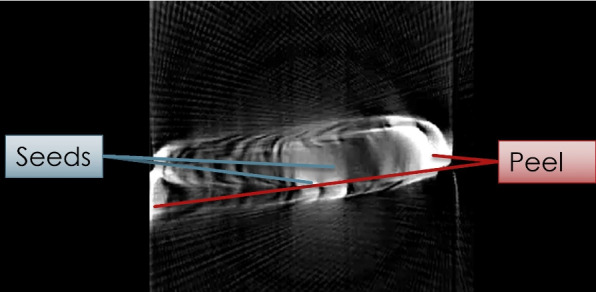
Reconstructed cross-sectional image of the lemon slice using inverse Radon transform. High-density features such as the peel and seeds appear as darker regions

These results validate the scanner’s ability to resolve internal variations in density, confirming the effectiveness of visible light transmission imaging for tomographic applications involving semitransparent objects. This is caused by denser regions attenuating more light, resulting in lower transmitted intensity and, therefore, darker pixels in the image. To further assess the grayscale contrast between key anatomical features, a quantitative region-of-interest (ROI) analysis was performed on the reconstructed image shown in Fig. 10. Representative ROIs were manually selected over the peel and seed regions. The peel exhibited a mean grayscale intensity of $0.99 \pm 0.02$ arbitrary units (AU), whereas the seeds showed a mean intensity of $0.71 \pm 0.04$ AU. This difference of 0.28 AU is statistically significant ($P< 0.001$), demonstrating the system’s ability to distinguish subtle internal structures based on optical attenuation contrast, despite their visual similarity in grayscale displays.

### 3D volume visualization

To further enhance interpretability and spatial context, the reconstructed slices were assembled into a 3D volume using the Vol3D toolbox in MATLAB. This step enabled volumetric rendering of the lemon slice, revealing a depth-dependent structure and the interrelationships between its internal components.

Adjustments to the transparency thresholds, lighting direction, and camera perspective in the Vol3D environment allowed for customized exploration of the volume. This rendering highlighted several structural features.

- The lemon’s outer peel is rendered with high opacity, emphasizing its circular boundary.
- Internal pulp is semi-transparent, providing contextual shape without occluding deeper features.
- Seeds appear as high-opacity inclusions, distinguishable due to their denser light-blocking properties.

**Fig. 11:**
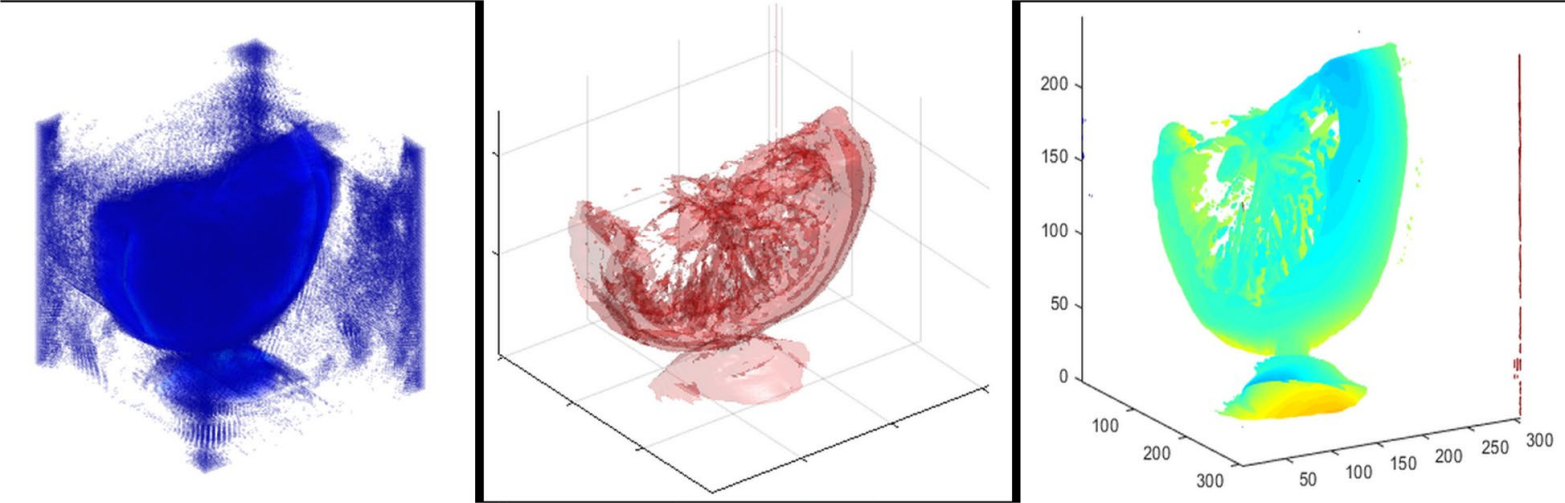
3D volume rendering of the lemon slice generated using Vol3D. Depth cues and transparency settings enable visualization of both surface and internal features

The successful generation of a high-fidelity 3D volume from 2D visible light projections demonstrates the effectiveness of the scanner pipeline, from hardware design to software rendering. This result confirms the system’s suitability for visualizing the internal composition of semitransparent specimens and supports its application in educational and exploratory imaging contexts. The final volume rendering is presented in Fig. 11.

## Discussion

The experimental results confirm that a low-cost portable OECT scanner based on visible light transmission can successfully reconstruct and visualize internal structures within semi-transparent specimens. This section reflects on the broader implications of the study, highlights its educational benefits, identifies its current limitations, and outlines directions for future development and extended applications.

### Advantages and educational value

The primary objective of this project was to design an imaging system that is technically functional, accessible, and pedagogically valuable. Unlike traditional CT systems that rely on ionizing radiation and require shielding, regulatory oversight, and specialized training, this optical scanner uses visible light, making it inherently safe and suitable for use in classrooms, laboratories, and informal learning environments.

The scanner’s design also provides an integrative learning platform across multiple disciplines.

- **Physics and optics:** Enables exploration of transmission, attenuation, and projection geometry.
- **Engineering and robotics:** Involves mechanical design, motor control, and hardware integration.
- **Computer science:** Requires microcontroller programming, data handling, and automation logic.
- **Mathematics:** Provides practical exposure to topics such as the Radon transform and inverse algorithms.

Its modular construction and the use of off-the-shelf components further support its role as an educational demonstrator that can be rebuilt, modified, and extended by students.

### Comparison with existing systems

This comparison in Table 2 highlights the distinct advantages of our educational OECT system. Although Zakariaee et al. [2] and Stavroulakis et al. [6] developed optical CT scanners for specialized applications in dosimetry and industrial inspection, our design prioritizes affordability, safety, and pedagogical accessibility. A key differentiator is the implementation of a complete, low-cost imaging pipeline from automated sinogram capture to 3D volumetric rendering, which is demonstrated using accessible biological specimens. Compared with the similar education-focused work of Lim et al. [1], our system provides a more integrated hardware and software solution with advanced 3D visualization capabilities while achieving a competitive balance of scanning speed of 200 s and spatial resolution of 0.1–0.2 mm. The use of consumer-grade components and open-source software creates a versatile and hands-on learning platform, making our system particularly suitable for teaching the core principles of tomography in STEM education.

**Table 2:** Comparison of our OECT system with existing systems

System	Estimated cost (USD)	Specimen type	Automation	Software	Scanning time	Spatial resolution
Lim et al. (2024) [1]	$250–$300	Lemon slice	Automated rotation + capture	MATLAB	$$\sim 200$$ s	$$\normalsize \sim 0.2 \,\textrm{mm}$$
Zakariaee et al. (2014) [2]	Not specified	Gel dosimeter	Motorized stage (1st Gen)	MATLAB	> 60 min	$$\sim$$ 1 mm
Stavroulakis et al. (2023) [6]	Not specified	Industrial glass, bottles	Full automation	Astra Toolbox	< 5 min	$$\sim$$ 0.5 mm
**Our system**	$705	Lemon slice	Automated rotation + capture	MATLAB	$$\sim$$ 200 s	$$\sim$$ 0.1–0.2 mm

### Reproducibility and experimental stability

To evaluate the reproducibility of the OECT system, multiple scan sessions were performed using lemon slices of comparable sizes and thicknesses under identical system configurations. Across repeated trials, the reconstructed images consistently revealed the primary anatomical features–peel, pulp, and seeds–with only minor variations in grayscale intensity levels and spatial sharpness.

The key factors influencing the reproducibility are as follows:

- **Specimen alignment:** Precise centering of the specimen on the rotation axis is critical. Even slight tilts or lateral displacements can introduce asymmetries in the sinogram, leading to artifacts in the reconstruction. To mitigate this, alignment guides and stabilizers (e.g., cork base with toothpick mount) were used during mounting.
- **Illumination stability:** The battery-powered LED backlight provided consistent output across sessions. The inclusion of a fixed diffuser panel ensured uniform lighting. However, significant ambient light or LED battery drain could degrade contrast; hence, scans were conducted in a fully enclosed box with fresh batteries before each session.
- **Mechanical jitter:** Since the rotation is motor-driven without feedback control, slight angular inconsistencies may occur. These were minimized by allowing the motor to settle before each capture and using friction-enhancing materials (e.g., paper towel) under the specimen to prevent slippage.
- **Camera settings:** All exposures were manually fixed using Program mode on the DSLR, and auto-focus was disabled to ensure consistent imaging parameters. No perceptible focus shift or exposure drift was observed between sessions.

### Limitations

Although the system demonstrates clear functionality, several inherent limitations should be acknowledged.

- **Specimen transparency:** The system depends on light transmission, restricting its use to objects that are at least partially transparent. Opaque or highly scattering specimens cannot be imaged effectively.
- **Limited performance with large or opaque specimens:** The system is optimized for thin slices like lemon sections. Whole fruit imaging, while technically feasible, suffers from limited light penetration through thicker tissues, resulting in low-contrast projections. Such scans could still demonstrate the fundamental trade-offs between optical and X-ray CT modalities.
- **Alignment sensitivity:** Accurate reconstruction depends on precise alignment of the specimen with the rotational axis and optical path. Misalignments introduce distortion and may degrade reconstruction quality.
- **Manual workflow:** While image capture is automated via interval shooting, other parts of the workflow, such as specimen alignment, mounting, and post-scan image formatting, remain manual and may introduce variability.
- **Lack of true depth resolution:** Unlike clinical CT, where physical slices are acquired layer by layer, the 3D volume in OECT is reconstructed mathematically from 2D projections without actual axial scanning.
- **Surface refraction artifacts:** Refraction at curved sample surfaces can create bright edge artifacts in reconstructed images. Optical CT systems typically use index-matching liquids to mitigate such effects; however, our educational design prioritizes simplicity and safety over quantitative accuracy, accepting refraction as an inherent limitation.

These constraints are common in first-generation demonstrator systems and guide future iterations.

### Opportunities for improvement

Several enhancements are envisioned to improve the system’s functionality, usability, and performance:

- **Automated stage and shutter control:** Integrating the camera and motor into a unified control system would synchronize frame capture with rotation, reducing timing errors and manual intervention.
- **Higher-speed components:** Employing faster image sensors and higher-precision motors would improve angular resolution and enable dynamic (time-lapse) OECT.
- **Precision mounting solutions:** Replacing the current tape-based specimen holder with magnetic or clamp-based fixtures would improve repeatability and reduce motion artifacts.
- **User interface and workflow automation:** Developing a graphical interface in MATLAB or Python could streamline preprocessing, reconstruction, and visualization, enabling non-programmers to operate the system effectively.
- **Collimated laser illumination:** Replacing the diffuse LED backlight with a collimated laser beam would provide a more accurate physical emulation of the line integrals required for the Radon transform. This design would significantly reduce light scattering within the specimen, leading to sharper reconstructions with better contrast and spatial resolution. It represents a key step from a qualitative demonstration towards a more quantitatively accurate educational tool.
- **Alternative imaging hardware:** While the DSLR provides high resolution, it dominates system costs. Replacing it with a low-cost industrial camera or webcam could further reduce expenses, trading some image quality for greater accessibility in educational settings.

These improvements would enhance performance and lower the barrier for wider adoption in educational and creative settings.

### Broader applicability

Although designed with education in mind, the scanner’s capabilities may extend to several domains that benefit from the noninvasive visualization of transparent or semi-transparent materials.

- **Industrial design and prototyping:** Inspection of transparent components, including 3D printed parts, molded plastics, and polymer-based assemblies.
- **Biological research:** Non-destructive analysis of plant tissues, fruit structures, and small soft-bodied organisms where internal layering is of interest.
- **Digital art and visualization:** Generation of 3D digital models for artistic exploration, physical-to-digital archiving, or immersive media applications.

These application areas illustrate the versatility of OECT in domains beyond clinical medicine, highlighting their potential for cross-disciplinary innovation in both STEM and creative disciplines.

## Conclusions

This study presents the design, implementation, and evaluation of a compact, low-cost OECT scanner for the visualization of semitransparent specimens. By leveraging visible light, a rotating stage, and a fixed imaging system enclosed within a light-isolated box, the scanner offers a safe, accessible, and replicable alternative to traditional X-ray-based CT systems, particularly in educational and demonstrative contexts.

The system combines the following mechanical, optical, and electronic subsystems: a 3D printed rotating platform powered by a stepper motor, Teensy-based microcontroller for motion control, and DSLR camera with a prime lens for high-quality image acquisition. The projection images were processed in MATLAB, where a sinogram was constructed, and an inverse Radon transform was applied to generate 2D reconstructions. They were further extended to 3D volume rendering using the Vol3D toolbox.

Validation experiments using a lemon slice confirmed the scanner’s ability to resolve key internal features such as the outer peel, internal pulp, and embedded seeds. Although the system is currently limited to transparent specimens and requires manual alignment and processing, its modular architecture and open-source design require further development and customization.

Overall, this project demonstrates the feasibility of adapting CT imaging principles to visible light and consumer-grade hardware, enabling new applications in STEM education, digital art, biological research, and industrial prototyping. Future enhancements, such as automated synchronization, improved mounting systems, and user-friendly software interfaces, may further expand the system’s accessibility and impact across disciplines.

## Data Availability

All the data and MATLAB code used in this study are available in Appendix A. Additional materials are available upon request. An instructional video was available at the https://www.youtube.com/watch?v=DR-WbpEjhc0.

## Appendix A: MATLAB codes for processing, reconstruction, and visualization

The following MATLAB scripts outline the complete workflow for loading the projection images, generating a sinogram, reconstructing a cross-sectional image, and visualizing the resulting volume.

## Appendix B: list of materials

Table 3 summarizes all key materials used in the construction of the optical CT scanner. Each component was selected based on accessibility, compatibility with the educational environment, and ease of integration. All items were either commercially available or easily fabricated using basic tools such as 3D printers and breadboards.

**Table 3:** List of materials used in the OECT scanner prototype

Material	Function
Plywood sheets	Construct the light-isolated enclosure that blocks ambient light and provides structural support for mounting subsystems
Milwaukee LED light bar	Serves as the backlight source for transmission imaging, delivering consistent and high-intensity illumination
Diffuser panel (acrylic or film)	Softens and evenly distributes light across the specimen to minimize shadows and improve contrast
PLA filament	Used in 3D printing to fabricate the rotating stage, camera rig, and motor mounts
NEMA 17 stepper motor	Controls the angular rotation of the specimen during scanning
A4988 stepper motor driver	Provides current regulation and step control to the motor from the microcontroller
Teensy 2.0 microcontroller	generates pulse sequences to drive the motor and synchronize movement with camera acquisition
Breadboard and jumper wires	Used for prototyping the control circuit without soldering; allows modular wiring
Nikon D5600 DSLR camera	Captures projection images with high resolution and dynamic range across all angular views
50 mm f/1.8 prime lens	Fixed-focus lens providing sharp, low-distortion imaging, ideal for close-range CT acquisition
Mounting materials (cork base, tape, toothpicks, screws)	Supports specimen attachment and alignment at the center of the rotating stage

## Appendix C: YouTube demonstration

A video demonstration showcasing the optical CT scanner setup, scanning process, and MATLAB-based reconstruction and rendering is available at the following link.

https://www.youtube.com/watch?v=DR-WbpEjhc0

The video includes:

- Overview of hardware setup and lighting enclosure
- Sinogram generation and cross-sectional reconstruction
- Interactive 3D rendering using Vol3D in MATLAB

## Abbreviations

ART: Algebraic reconstruction technique
AU: Arbitrary units
CR: Cinematic rendering
CT: Computed tomography
DSLR: Digital single-lens reflex
FBP: Filtered backprojection
LED: Light emitting diode
OECT: Optically emulated computed tomography
OCT: Optical coherence tomography
OPT: Optical projection tomography
PLA: Polylactic acid
ROI: Region-of-interest
USB: Universal serial bus
Vol3D: Volumetric 3D MATLAB toolbox
vis-OCT: Visible-light optical coherence tomography
